# Recent Advances in Gestational Diabetes Mellitus

**DOI:** 10.3390/jcm10102202

**Published:** 2021-05-19

**Authors:** Katrien Benhalima

**Affiliations:** Department of Endocrinology, University Hospital Gasthuisberg, KU Leuven, Herestraat 49, 3000 Leuven, Belgium; katrien.benhalima@uzleuven.be

The incidence of gestational diabetes mellitus (GDM) and overt diabetes in pregnancy is rising globally. GDM leads to increased risks for maternal and neonatal adverse pregnancy outcomes. In addition, GDM is also associated with an increased long-term metabolic risk in mothers and offspring [1]. Although much is known about GDM, evidence gaps persist. For instance, more research is needed on how to prevent GDM, on whether screening and treatment of GDM in early pregnancy are beneficial, on non-fasting biomarkers to screen for GDM, on new biomarkers to predict pregnancy complications, and on how to reduce the long-term metabolic risk in mothers and infants after delivery. To address this important health issue, the present Special Issue in the Journal of Clinical Medicine was dedicated to recent advances in the field of GDM. This Special Issue published 16 articles on this topic. The issues evaluated in these papers varied from research on pregnancy outcomes to screening and diagnosis of GDM, use of new biomarkers, and evaluation of long-term metabolic risk and intervention strategies postpartum in mothers and offspring. 

This Special Issue includes various important findings. For example, Zawiejska et al. show that in high-risk women, early screening for GDM using the IADPSG criteria may be a useful predictor of congenital anomalies [2]. Another study showed that inadequate gestational weight gain was very common in a large cohort of GDM with a lower risk for adverse pregnancy outcomes in women with insufficient gestational weight gain [3]. Another study demonstrated that third-trimester fetal anthropometric parameters such as fetal weight centile can be used to predict adverse neonatal outcomes beyond the classical maternal risk factors [4]. The ELENA study showed that multidisciplinary group education might help to better manage the increasing workload and is also positively evaluated by women with GDM [5].

There has long been controversy on how to screen and diagnose GDM. Bogdanet et al. provide an extensive narrative review on the several factors that can influence the results of an oral glucose tolerance test (OGTT) in pregnancy and negatively impact patient care [6]. Another review discusses emerging biomarkers to simplify screening for GDM without the need for an OGTT [7]. Raets, L. et al. reviewed the current evidence and controversies on screening for GDM in early pregnancy [8]. Another important evidence gap is how to screen and diagnose GDM in pregnant women with a history of bariatric surgery. Deleus, E. et al. reviewed the challenges of using an OGTT in pregnancy after bariatric surgery and discussed potential alternatives to screen for GDM in this high-risk population. In addition, the evidence on the association between hypoglycemia and glycemic variability with adverse pregnancy outcomes in this population was reviewed [9]. A large retrospective French study evaluated different screening strategies for hyperglycemia in pregnancy to limit the number of OGTTs during pandemics [10]. Egan, A.M. et al. from the Mayo Clinic provided an extensive narrative review on the recurrence of GDM in a second pregnancy and presented the experience from their center [11]. 

Deischinger, C. et al. evaluated secretagogin (SCGN) in pregnancy, which is a calcium-binding protein related to insulin release in the pancreas. They showed that SCGN is related to insulin secretion but is unrelated to the diagnosis of GDM [12]. The study design and rationale were presented of the PROMIS study, a Dutch multi-center longitudinal study to evaluate insulin sensitivity and glucose metabolism in overweight pregnant women and the impact on pregnancy outcomes, including early growth of the offspring [13]. In addition, a population-based study evaluated the evolution in the prevalence of diabetes among twin pregnancies and pregnancy complications in Catalonia [14]. 

Long-term follow-up after pregnancy with GDM is important to timely prevent metabolic complications in mothers and offspring. The study protocol of the Melinda trial was presented. This is an ongoing RCT comparing a telephone- and mobile-based lifestyle intervention with standard of care in women with prediabetes after GDM [15]. Two-year follow-up data of offspring of the San Carlos GDM Prevention Study showed that a Mediterranean diet during pregnancy was associated with a reduction in hospitalizations with antibiotic and corticosteroid treatment, and fewer hospitalizations due to asthma/bronchiolitis [16]. A review with extensive scope on the long-term metabolic risk in the offspring showed that the prevalence of overweight/obesity and glucose disorders is higher in offspring exposed to GDM compared to unexposed offspring. Importantly, overall, this association remained significant after correction for maternal overweight [17].
